# Catalogue of type specimens deposited in the Herpetology Collection of the Natural History Museum Gustavo Orcés V. at Escuela Politécnica Nacional (Ecuador)

**DOI:** 10.3897/BDJ.11.e108596

**Published:** 2023-08-29

**Authors:** Ana Almendáriz, Diego Almeida-Reinoso, Monica A. Guerra

**Affiliations:** 1 Laboratorio de Herpetología, Departamento de Biología, Escuela Politécnica Nacional, Quito, Ecuador Laboratorio de Herpetología, Departamento de Biología, Escuela Politécnica Nacional Quito Ecuador

**Keywords:** Amphibia, Reptilia, Ecuador, type specimens, MEPN-H, *Pristimantis*, *Atelopus*, *Atractus*, *Enyaloides*

## Abstract

**Background:**

The Herpetology Collection of the Natural History Museum Gustavo Orcés V. at Escuela Politécnica Nacional (MEPN-H) in Quito maintains more than sixteen thousand curated specimens and it comprises Ecuador ´s second largest collection of herps.

**New information:**

The Collection contains 193 type specimens: 14 holotypes, 34 paratopotypes and 145 paratypes, which correspond to 10 families, 17 genera and 32 species. The collection of type specimens is particularly important in the genera *Atelopus* and *Pristimantis* in amphibians and the genera *Atractus* and *Enyaloides* in reptiles. An assessment of the geographic distribution showed that collection sites of type specimens are clustered towards the south of Ecuador in the provinces of Zamora Chinchipe, Morona Santiago and Pastaza in the Amazon Region; and in the provinces of Carchi and Azuay in the Andes. The collection of type specimens dates from 1955 to 2013, comprising an invaluable source of historical biodiversity data.

## Introduction

The Natural History Museum Gustavo Orcés V. at Escuela Politécnica Nacional (MEPN) was established as the Museum of Zoology of Escuela Politécnica Nacional in 1946 (Ortega et al. 2014). It was founded by Robert Hoffstetter and Gustavo Orcés, with collections made by Franz Spillmann and Manuel Olalla from 1930 (Hoffstetter 1952). Since then, until the early 1980s, all the zoological collections in the Museum expanded primarily through contributions from the Olalla brothers and Luis Abuja (Albuja et al. 1980, Albuja 1999). Currently, MEPN houses six biological scientific collections, namely Paleontology, Ictiology, Mastozoology, Ornitology, Invertebrates and Herpetology.

The Herpetology Collection of the Natural History Museum Gustavo Orcés at Escuela Politécnica Nacional (MEPN-H), started as a section of the Museum in 1983, with Ana Almendáriz serving as its first curator. Over time, the MEPN-H collection increased through contributions from its former curator (Almendáriz et al. 2014, Almendáriz et al. 2017), as well as national and international researchers, students and volunteers. To date, the Herpetology Collection holds 5402 reptile specimens and 10907 amphibian specimens. The first specimen from Ecuador, deposited in MEPN-H, dates back to the 1930s and corresponds to an individual of *Siphonopsannulatus* (Order Gymnophiona).

In 1955, MEPN-H received its first set of type specimens. These initial type specimens consist of paratypes of *Holcosusorcesi* (Teiidae) and *Atractusgaigae* (Colubridae), which likely constitute the most ancient reptilian types deposited in an Ecuadorian Natural History Museum. It is noteworthy that, during the 1950s, specimens were acquired by the Museum through various means, including purchases and donations. The MEPN-H holds unique historical biodiversity data, as it also includes some of the oldest amphibian type specimens deposited in a Natural History Museum in Ecuador, the paratypes of *Caeciliadisossea*, collected around 1968 (Taylor 1968).

## Sampling methods

### Study extent

The Herpetology Collection of the Natural History Museum Gustavo Orcés at Escuela Politécnica Nacional (MEPN-H) encompasses specimens from all 24 provinces in Ecuador, including the Galapagos Islands. This comprehensive collection encompasses a total of 193 type specimens, consisting of 14 holotypes, 34 paratopotypes and 145 paratypes, representing 10 families, 17 genera and 32 species (Suppl. material 1).

Within this collection, there are 164 amphibians and 29 reptiles. In the order Anura, MEPN-H holds 10 holotypes, 34 paratopotypes and 114 paratypes. In the order Gymnophiona, there are six paratypes, while the order Squamata consists of four holotypes and 25 paratypes (refer to Table 1 for further details).

Amongst the type specimens, certain genera stand out for their significant representation. Particularly, the genus *Atelopus* and *Pristimantis* are highly abundant in amphibians, while the genus *Atractus* and *Enyaloides* dominate for reptiles. Notably, years with the highest number of collected type specimens include 1986 and 2012, which yielded new records of *Atelopus* in Carchi and *Pristimantis* in Morona Santiago and Zamora Chinchipe.

### Sampling description

Sampling methods vary amongst collectors and projects. Most of them include Visual Encounter Surveys (VES), sampling plots or linear transects (Angulo et al. 2006). Type specimens deposited in the Herpetology Collection MEPN-H are the result of various donations, purchases and research projects. Before the 1980s, all specimens in the collection were preserved in formalin 10%. During the first years of the 80s, wet specimens were transferred to ethanol 70%. Most of amphibian and reptile type specimens collected after the 80s were fixed with formalin 10% and preserved in ethanol 70%, except for MEPN-H 14219 which corresponds to a cleared and stained specimen stored in glycerine.

### Quality control

The consistency of data on type-specimen records was verified by comparisons with the information in original publications of the corresponding new species. Records were exported as a Darwin Core Archive file which was uploaded to the IPT (Integrated Publishing Toolkit) hosted by Ministerio de Ambiente, Agua y Transición Ecológica del Ecuador (http://biodiversidad.ambiente.gob.ec:8099/biodiversidad-web/inicio).

Nomenclature follow Frost (2023) for amphibians and Uetz et al. (2023) for reptiles.

### Step description

The data validation and curation process commenced with an extensive search for scientific descriptions pertaining to herpetological type specimens deposited at Escuela Politecnica Nacional. Collection ID: MEPN-H has been established since 2022, but previous years specimens were referred to as EPN or MEPN. We compared the specimen details mentioned in the corresponding scientific papers with our collection database, field notes if available and the physical specimens housed in our collection.

The majority of type specimens exhibited consistency between the descriptions in the papers, our database and their physical counterparts within our collection. However, during this rigorous validation process, we encountered three specimens that were found to be physically absent from our collection. These specimens are MEPN-H 1226 (*Atelopuspodocarpus*), MEPN-H 13680 (*Chiasmocleisparkeri*) and MEPN-H 14445 (*Pristimantistinguichaca*).

In addition, we identified two instances where the corresponding publication erroneously referenced specimen numbers. Specifically, MEPN-H 11411 was mistakenly referred to as EPN 1141, while MEPN-H 12311 was incorrectly mentioned as EPN 1231 (Almendáriz et al. 2012). Both specimens correspond to *Excidobatescondor*.

Furthermore, we discovered three specimens of *Anolisvanzolinii* that were referenced in the corresponding papers with field numbers instead of the MEPN-H number. In Williams et al. (1996), EPN 2218-9 and EPN 2221 actually correspond to MEPN-H 9823-5, respectively.

## Geographic coverage

### Description

**Figure 1.**
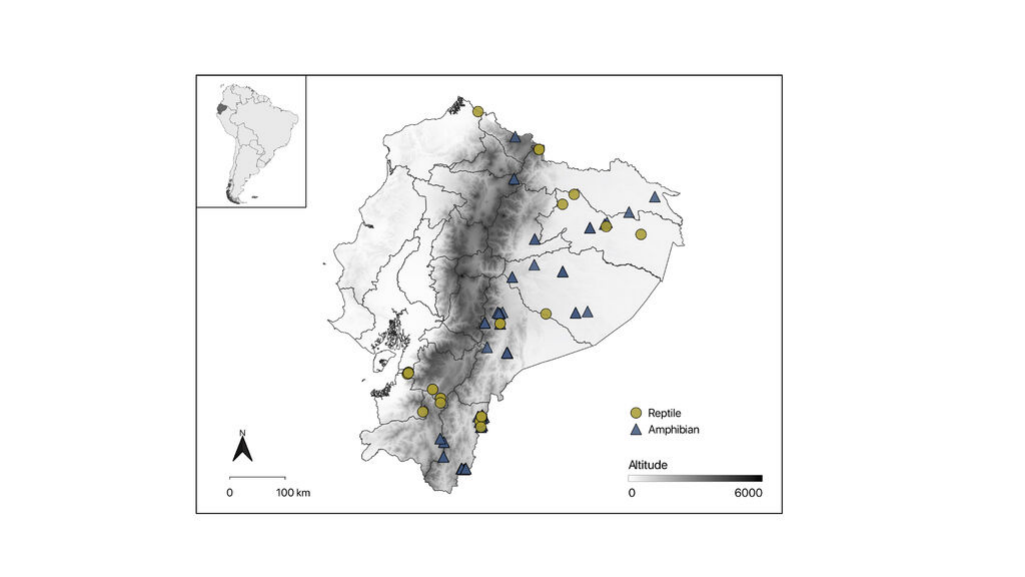
Localities of type specimens deposited at MEPN-H.

Collection sites of type specimens are clustered towards the south of Ecuador in the provinces of Zamora Chinchipe, Morona Santiago and Pastaza in the Amazon Region and in the provinces of Carchi and Azuay in the Andes (Fig. 1). Localities with the highest number of types include Tinguichaca, Paquisha and Cerro Plateado.

### Coordinates

-4.61 S and 1.20 N Latitude; -79.70 W and -75.67 W Longitude.

## Taxonomic coverage

### Description

Corresponding protologues in Suppl. materials 1, 2.

### Taxa included

**Table:** 

Rank	Scientific Name	
kingdom	Animalia	
subkingdom	Eumetazoa	
phylum	Chordata	
subphylum	Vertebrata	
class	Amphibia	
class	Reptilia	
order	Anura	
order	Squamata	
order	Gymnophiona	
family	Bufonidae	
family	Caeciliidae	
family	Colubridae	
family	Dendrobatidae	
family	Gymnophthalmidae	
family	Hylidae	
family	Iguanidae	
family	Microhylidae	
family	Strabomantidae	
family	Teiidae	
species	*Andinosauraaurea*	
species	*Anolispodocarpus*	
species	*Anolisvanzolinii*	
species	*Atelopuspastuso*	
species	*Atelopuspodocarpus*	
species	*Atractusatlas*	
species	*Atractusgaigeae*	
species	*Atractuspachacamac*	
species	*Boanaalmendarizae*	
species	*Boanamaculateralis*	
species	*Caeciliadisossea*	
species	*Chiasmocleisparkeri*	
species	*Dipsasjamespetersi*	
species	*Dipsasoligozonata*	
species	*Echinosauraorcesi*	
species	*Enyalioidesrubrigularis*	
species	*Enyalioidestouzeti*	
species	*Excidobatescondor*	
species	*Holcosusorcesi*	
species	*Hyloscirtuscondor*	
species	*Noblellapersonina*	
species	*Osornophrynepuruanta*	
species	*Osteocephaluscannatellai*	
species	*Osteocephalusfuscifacies*	
species	*Pristimantisalmendariz*	
species	*Pristimantisbarrigai*	
species	*Pristimantischuruwiai*	
species	*Pristimantislatericius*	
species	*Pristimantismuranunka*	
species	*Pristimantispaquishae*	
species	*Pristimantissambalan*	
species	*Pristimantistinguichaca*	

## Temporal coverage

### Notes

Specimens of *C.disossea* may have been collected before 1968. Specimen of *A.gaigeae* has a possible collection date of 1955.

**Figure 2.**
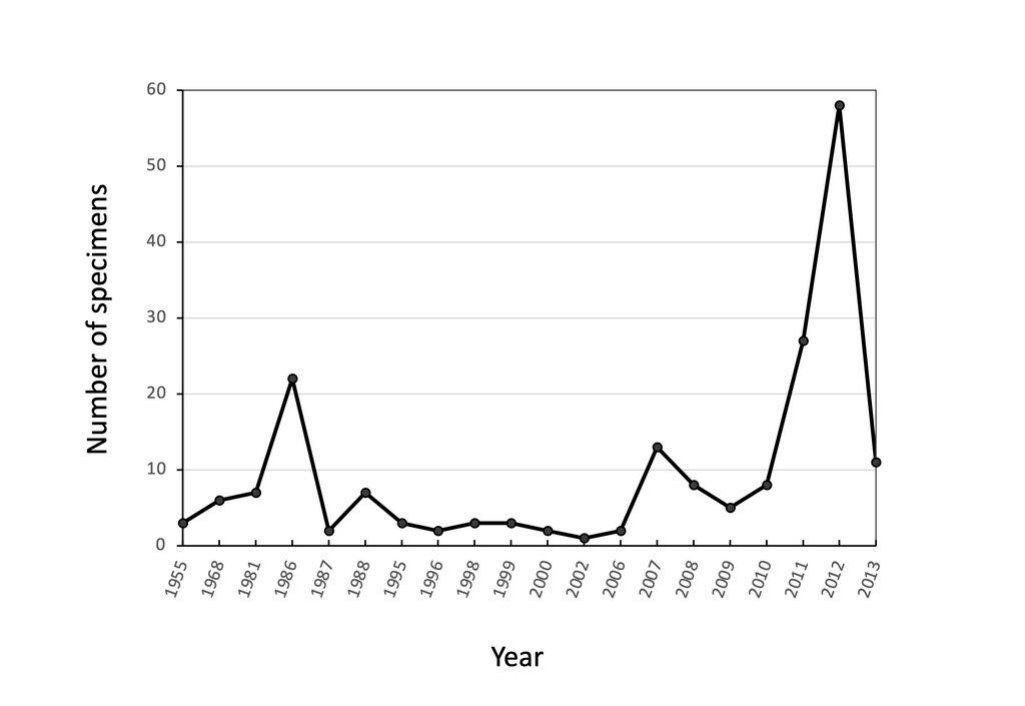
Number of type specimens per year deposited at MEPN-H.

DATA RANGE: 1955–2013 (Fig. 2).

## Collection data

### Collection name

Herpetology Collection of the Natural History Museum Gustavo Orcés V.- Escuela Politécnica Nacional

### Collection identifier

MEPN-H

### Parent collection identifier

MEPN

### Specimen preservation method

Entire specimens ethanol 70%, tissue samples ethanol 95%, cleared and stained specimen glycerine.

## Usage licence

### Usage licence

Other

### IP rights notes

Creative Commons Attribution (CC-BY) 4.0.

## Data resources

### Data package title

Catalogue of type specimens deposited in the Herpetology Collection of the Natural History Museum Gustavo Orcés V. at Escuela Politécnica Nacional (Ecuador)

### Number of data sets

1

### Data set 1.

#### Data set name

Catalogue of type specimens deposited in the Herpetology Collection of the Natural History Museum Gustavo Orcés V. at Escuela Politécnica Nacional (Ecuador)

#### Data format

Darwin Core Archive

#### Character set

UTF-8

#### Download URL

https://www.gbif.org/dataset/e6459139-bddc-4f60-9f4e-957380450ee8

#### Data format version

1.3

#### Description

Dataset contains 193 type specimens: 14 holotypes, 34 paratopotypes and 145 paratypes, corresponding to 10 families, 17 genera and 32 species (Guerra and Almendáriz 2023). Column labels follow Darwin Core Maintenance Group (2021).

**Data set 1.** 

Column label	Column description
occurrenceID	An identifier for the Occurrence. Record in the dataset or collection.
type	The nature of the presence record, taxon or event.
language	A language of the resource.
licence	A legal document giving official permission to do something with the resource.
rightsHolder	A person or organisation owning or managing rights over the resource.
accessRights	Information about who can access the resource or an indication of its security status.
institutionID	An identifier for the institution having custody of the object(s) or information referred to in the record.
collectionID	An identifier for the collection or dataset from which the record was derived.
datasetID	An identifier for the set of data. May be a global unique identifier or an identifier specific to a collection or institution.
institutionCode	The name (or acronym) in use by the institution having custody of the object(s) or information referred to in the record.
collectionCode	The name, acronym, coden or initialism identifying the collection or dataset from which the record was derived.
datasetName	The name identifying the dataset from which the record was derived.
ownerInstitutionCode	The name (or acronym) in use by the institution having ownership of the object(s) or information referred to in the record.
basisOfRecord	The specific nature of the data record.
catalogNumber	An identifier (preferably unique) for the record within the dataset or collection.
recordedBy	A list (concatenated and separated) of names of people, groups or organisations responsible for recording the original Occurrence.
disposition	The current state of a specimen with respect to the collection identified in collectionCode or collectionID.
year	The four-digit year in which the Event occurred, according to the Common Era Calendar.
month	The integer month in which the Event occurred.
day	The integer day of the month on which the Event occurred.
eventDate	The date-time or interval during which an Event occurred.
continent	The name of the continent in which the Location occurs.
country	The name of the country or major administrative unit in which the Location occurs.
countryCode	The standard code for the country in which the Location occurs.
stateProvince	The name of the next smaller administrative region than country (state, province, canton, department, region etc.) in which the Location occurs.
locality	The specific description of the place.
minimumElevationInMetres	The lower limit of the range of elevation (altitude, usually above sea level), in metres.
maximumElevationInMetres	The upper limit of the range of elevation (altitude, usually above sea level), in metres.
decimalLatitude	The geographic latitude (in decimal degrees, using the spatial reference system given in geodeticDatum) of the geographic centre of a Location. Positive values are north of the Equator, negative values are south of it. Legal values lie between -90 and 90, inclusive.
decimalLongitude	The geographic longitude (in decimal degrees, using the spatial reference system given in geodeticDatum) of the geographic centre of a Location. Positive values are east of the Greenwich Meridian, negative values are west of it. Legal values lie between -180 and 180, inclusive.
geodeticDatum	The ellipsoid, geodetic datum or spatial reference system (SRS), upon which the geographic coordinates given in decimalLatitude and decimalLongitude are based.
typeStatus	A list (concatenated and separated) of nomenclatural types (type status, typified scientific name, publication) applied to the subject.
taxonID	An identifier for the set of taxon information (data associated with the Taxon class). May be a global unique identifier or an identifier specific to the dataset.
scientificName	An identifier for the nomenclatural (not taxonomic) details of a scientific name.
kingdom	The full scientific name of the kingdom in which the taxon is classified.
phylum	The full scientific name of the phylum or division in which the taxon is classified.
class	The full scientific name of the class in which the taxon is classified.
order	The full scientific name of the order in which the taxon is classified.
family	The full scientific name of the family in which the taxon is classified.
genus	The full scientific name of the genus in which the taxon is classified.
specificEpithet	The name of the first or species epithet of the scientificName.
taxonRank	The taxonomic rank of the most specific name in the scientificName.
scientificNameAuthorship	The authorship information for the scientificName formatted according to the conventions of the applicable nomenclaturalCode.
preparations	A preparation or preservation method for a specimen.

## Supplementary Material

4AE213D3-7770-5AC3-9874-BAF7655D3D34 10.3897/BDJ.11.e108596.suppl1 Supplementary material 1 Dataset of type specimens MEPN-H Data type ocurrences File: oo_868367.txt https://binary.pensoft.net/file/868367 A. Almendáriz, D. Almeida-Reinoso, M. A. Guerra

E10C8045-94A3-5743-BE70-5A9929897000 10.3897/BDJ.11.e108596.suppl2 Supplementary material 2 Protologues of type specimens MEPN-H Data type list of references File: oo_865262.pdf https://binary.pensoft.net/file/865262 A. Almendáriz, D. Almeida-Reinoso, M.A. Guerra

## Figures and Tables

**Table 1.** Species of type specimens deposited at MEPN-H.

**Species**	**HOLOTYPE**	**PARATOPOTYPE**	**PARATYPE**	**Total**
**Order**: Anura	10	34	114	158
*Atelopuspastuso*			18	18
*Atelopuspodocarpus*			9	9
*Boanaalmendarizae*			5	5
*Boanamaculateralis*			4	4
*Chiasmocleisparkeri*	1	1	11	13
*Excidobatescondor*	1		9	10
*Hyloscirtuscondor*	1	7	1	9
*Noblellapersonina*	1		6	7
*Osornophrynepuruanta*			3	3
*Osteocephaluscannatellai*			6	6
*Osteocephalusfuscifacies*			2	2
*Pristimantisalmendariz*	1		3	4
*Pristimantisbarrigai*	1	1		2
*Pristimantischuruwiai*			4	4
*Pristimantislatericius*	1	5		6
*Pristimantismuranunka*	1		16	17
*Pristimantispaquishae*	1	10		11
*Pristimantissambalan*		2		2
*Pristimantistinguichaca*	1	8	17	26
**Order**: Gymnophiona			6	6
*Caeciliadisossea*			6	6
**Order**: Squamata	4		25	29
*Andinosauraaurea*			1	1
*Anolispodocarpus*			2	2
*Anolisvanzolinii*			3	3
*Atractusatlas*	1			1
*Atractusgaigeae*			1	1
*Atractuspachacamac*			6	6
*Dipsasjamespetersi*	1		2	3
*Dipsasoligozonata*	1			1
*Echinosauraorcesi*			1	1
*Enyalioidesrubrigularis*			3	3
*Enyalioidestouzeti*	1		4	5
*Holcosusorcesi*			2	2
**Total**	**14**	**34**	**145**	**193**
