# Supplementary material for: Catalogue of type specimens deposited in the Herpetology Collection of the Natural History Museum Gustavo Orcés V. at Escuela Politécnica Nacional (Ecuador)
Source: Biodivers Data J. 2023 Aug 29;11:e108596. doi: 10.3897/BDJ.11.e108596 (PMC10481154; doi:10.3897/BDJ.11.e108596)
Supplement: Supplementary material 2 — Protologues of type specimens MEPN-H [file bdj-11-e108596-s002.pdf]

Protologues of type specimens MEPN-H

- Almendáriz A, Ron S, Brito J (2012) Una especie nueva de rana venenosa de altura del género *Excidobates* (Dendrobatoidea: Dendrobatidae) de la Cordillera del Cóndor. Papéis Avulsos de Zoologia 52 (32): 387-399. https://doi.org/10.1590/ s0031-10492012021200001
- Almendáriz A, Brito J, Batallas D, Ron S (2014) Una especie nueva de rana arbórea del género *Hyloscirtus* (Amphibia: Anura: Hylidae) de la Cordillera del Cóndor. Papéis Avulsos de Zoologia 54 (4): 33-49. https://doi.org/10.1590/0031-1049.2014.54.04
- Almendáriz A, Brito J, Batallas D, Vaca-Guerrero J, Ron S (2017) Una especie nueva de rana del género *Chiasmocleis* (Microhylidae: Gastrophryninae) de la Cordillera del Cóndor, Ecuador. Papéis Avulsos de Zoologia 57 (10): 119-136. https://doi.org/ 10.11606/0031-1049.2017.57.10
- Ayala-Varela F, Torres-Carvajal O (2010) A new species of dactyloid anole (Iguanidae, Polychrotinae, *Anolis*) from the southeastern slopes of the Andes of Ecuador. ZooKeys 53: 59-73. https://doi.org/10.3897/zookeys.53.456
- Batallas D, Brito J (2014) Nueva especie de rana del género *Pristimantis* del grupo lacrimosus (Amphibia: Craugastoridae) del Parque Nacional Sangay, Ecuador. Papéis Avulsos de Zoologia 54 (5): 51-62. https://doi.org/10.1590/0031-1049.2014.54.05
- Brito J, Pozo-Zamora G (2013) Una nueva especie de rana terrestre del género *Pristimantis* (Amphibia: Craugastoridae), de la Cordillera de Kutukú, Ecuador. Papéis Avulsos de Zoologia 53 (24): 315-325. https://doi.org/10.1590/ s0031-10492013002400001
- Brito J, Batallas D, Velalcázar D (2014) Nueva especie de rana terrestre del género *Pristimantis* (Amphibia: Craugastoridae), meseta de la Cordillera del Cóndor. Papéis Avulsos de Zoologia 54 (30): 435-466. https://doi.org/10.1590/0031-1049.2014.54.30
- Brito J, Ojala-Barbour R, Batallas D, Almendáriz A (2016) A New Species of *Pristimantis* (Amphibia: Strabomantidae) from the Cloud Forest of Sangay National Park, Ecuador. Journal of Herpetology 50 (2): 337-344. https://doi.org/10.1670/13-103
- Brito J, Batallas D, Yánez-Muñoz M (2017a) Ranas terrestres *Pristimantis* (Anura: Craugastoridae) de los bosques montanos del río Upano, Ecuador: Lista anotada, patrones de diversidad y descripción de cuatro especies nuevas. Neotropical Biodiversity 3 (1): 125-156. https://doi.org/10.1080/23766808.2017.1299529
- Brito J, Almendáriz A, Batallas D, Ron S (2017b) Nueva especie de rana bromelícola del género *Pristimantis* (Amphibia: Craugastoridae), meseta de la Cordillera del Cóndor, Ecuador. Papéis Avulsos de Zoologia 57 (15): 177-195. https://doi.org/ 10.11606/0031-1049.2017.57.15
- Brito J, Almendáriz A (2018) Una especie nueva de rana *Pristimantis* (Amphibia: Strabomantidae) de ojos rojos de la Cordillera de Cóndor, Ecuador. Cuadernos de Herpetología 32 (1): 31-40. https://doi.org/10.31017/cdh.2018
- Caminer M, Ron S (2014) Systematics of treefrogs of the *Hypsiboas calcaratus* and *Hypsiboas fasciatus* species complex (Anura, Hylidae) with the description of four new species. ZooKeys 370: 1-68. https://doi.org/10.3897/zookeys.370.6291
- Coloma L, Duellman W, Almendáriz A, Ron S, Terán-Valdéz A, Juan G (2010) Five new (extinct?) species of *Atelopus* (Anura: Bufonidae) from Andean Colombia, Ecuador, and Peru. Zootaxa 2574: 1-54. https://doi.org/10.11646/zootaxa.2574.1.1
- Fritts T, Almendáriz A, Samec S (2002) A New Species of *Echinosaura* (Gymnophthalmidae) from Ecuador and Colombia with Comments on Other Members of the Genus and *Teuchocercus keyi*. Journal of Herpetology 36 (3): 349-355. https:// doi.org/10.1670/0022-1511(2002)036[0349:ansoeg]2.0.co;2
- Gluesenkamp A, Guayasamín J (2008) A new species of *Osornophryne* (Anura: Bufonidae) from the Andean highlands of northern Ecuador. Zootaxa 1828: 18-28.
- Harvey M, Almendáriz A, Brito J, Batallas D (2013) A new species of *Noblella* (Anura: Craugastoridae) from the Amazonian Slopes of the Ecuadorian Andes with Comments on *Noblella lochites* (Lynch). Zootaxa 3635 (1): 1-14. https://doi.org/10.11646/zootaxa. 3635.5
- Jungfer K, Ron S, Seipp R, Almendáriz A (2000) Two new species of hylid frogs, genus *Osteocephalus*, from Amazonian Ecuador. Amphibia-Reptilia 21: 327-340.

- Melo-Sampaio P, Passos P, Prudente A, Venegas P, Torres-Carvajal O (2021) Systematic review of the polychromatic ground snakes *Atractus snethlageae* complex reveals four new species from threatened environments. Journal of Zoological Systematics and Evolutionary Research 59 (3): 718-747. https://doi.org/10.1111/jzs. 12453
- Orcés G, Almendáriz A (1989) Presencia en el Ecuador de los Colúbridos del Género *Sibynomorphus*. Politécnica 14 (2): 57-67.
- Passos P, Scanferla A, Melo-Sampaio P, Brito J, Almendariz A (2018) A giant on the ground: another large-bodied *Atractus* (Serpentes: Dipsadinae) from Ecuadorian Andes, with comments on the dietary specializations of the goo-eaters snakes. Anais da Academia Brasileira de Ciências 91: 1-14. https://doi.org/ 10.1590/0001-3765201820170976
- Peters J (1964) The Lizard genus *Ameiva* in Ecuador. *Bulletin of the Southern California Academy of Sciences* 63 (3): 113-127.
- Ron S, Venegas P, Toral E, Read M, Ortiz D, Manzano A (2012) Systematics of the *Osteocephalus buckleyi* species complex (Anura, Hylidae) from Ecuador and Peru. ZooKeys 229: 1-52. https://doi.org/10.3897/zookeys.229.3580
- Sánchez-Pacheco S, Aguirre-Peñafiel V, Torres-Carvajal O (2012) Lizards of the Genus *Riama* (Squamata: Gymnophthalmidae): The Diversity in Southern Ecuador Revisited. South American Journal of Herpetology 7 (3): 259-275. https://doi.org/ 10.2994/057.007.0308
- Savage J (1955) Descriptions of new colubrid snakes, genus *Atractus*, from Ecuador. Proceedings of the Biological Society of Washington 68: 11-20. URL: https://biostor.org/ reference/65649
- Taylor E (1968) The Caecilians of the World: A Taxonomic Review. University of Kansas Press 848.
- Torres-Carvajal O, Almendáriz A, Valencia J, Yánez-Muñoz M, Reyes J (2008) A new species of *Enyalioides* (Iguanidae: Hoplocercinae) from southwestern Ecuador. Papéis Avulsos de Zoologia 48 (20): 227-235. https://doi.org/10.1590/s0031-10492008002000001
- Torres-Carvajal O, de Queiroz K, Etheridge R (2009) A new species of iguanid lizard (Hoplocercinae, Enyalioides) from southern Ecuador with a key to eastern Ecuadorian *Enyalioides*. ZooKeys 27: 59-71. https://doi.org/10.3897/zookeys.27.273
- Williams E, Orcés G, Matheus J, Bleiweiss R (1996) A new giant phenacosaur (Squamata: Iguania). Breviora 505: 1-33. URL: https://biostor.org/reference/4049
